# Supplementary material for: SpeS: A Novel Superantigen and Its Potential as a Vaccine Adjuvant against Strangles
Source: Int J Mol Sci. 2020 Jun 23;21(12):4467. doi: 10.3390/ijms21124467 (PMC7352279; doi:10.3390/ijms21124467)
Supplement: Supplementary file 1 [file ijms-21-04467-s001.pdf]

International Journal of *Molecular Sciences*

MDPI

## Supplementary Materials

**Table S1.** Primers used for cloning and sequencing

| | | **PRIMERS SEQUENCE** [1,2] |
|---|---|---|
| **PGEX3** | | 5′ GGGCTGCAAGCCACGTTTGGTG |
| | | 5′ CCGGGAGCTGCATGTGTCAGAGG |
| ***SPES*** | *speS* | 5′ CTACAAAGGTGGTAGGATCG |
| | | 5’ CGATCCTACCACCTTTGTAG |
| | Y39I SDM* | 5′ AAAAGTAAACTGCTTATTGCATACACTATAAC |
| | | 5′ GTTATAGTGTATGCAATAAGCAGTTTACTTTT |
| **PRIMERS FOR RECOMBINANT FUSION PROTEINS** | | |
| **START OF *SPES*** | | 5′ GACGACGGATCCTTGTCAATAGTGAATCCCACAAAG |
| **SEQ_0402** | SEQ_0402 | 5′ GTCGTCGGATCCTTGCGACTACCCTAGCAGGACAAAC |
| | SEQ_0402$^{ala}$ | 5′ GATGTTTATCTTGAAAAAGCAGCGGCAGCAGCGACTACCCTAGCAGGACAAAC |
| | | 5′ CTAGGGTAGTCGCTGCTGCCGCTGCTTTTTCAAGATAAACATCAAAATGGC |
| | SEQ_0402$^{GPGP}$ | 5′ GATGTTTATCTTGAAAAAGGTCCTGGTCCTGCGACTACCCTAGCAGGACAAAC |
| | | 5′ CTAGGGTAGTCGCAGGACCAGGACCTTTTTCAAGATAAACATCAAAATGGC |
| | SEQ_0402 cloning primer | 5′ GACGACGAATTCTGATGGAAGTGATTGCTCAGG |
| **SEQ_1959** | SEQ_1959 | 5′ GTCGTCGGATCCTTAATACCTATACGACTTATGCCAG |
| | SEQ_1959$^{ala}$ | 5′ GATGTTTATCTTGAAAAAGCAGCGGCAGCAAATACCTATACGACTTATGCCAG |
| | | 5′ CGTATAGGTATTTGCTGCCGCTGCTTTTTCAAGATAAACATCAAAATGGC |
| | SEQ_1959$^{GPGP}$ | 5′ GATGTTTATCTTGAAAAAGGTCCTGGTCCTAATACCTATACGACTTATGCCAG |
| | | 5′ CGTATAGGTATTAGGACCAGGACCTTTTTCAAGATAAACATCAAAATGGC |
| | SEQ_1959 cloning primer | 5′ GTCGTCGAATTCAGATCTTGGCAAAGCCTTAGTC |
| **SEQ_0933** | SEQ_0933 | 5′ GTCGTCGGATCCTTGATTCTGTTGAGTCAGCTGG |
| | SEQ_0933$^{ala}$ | 5′ GATGTTTATCTTGAAAAAGCAGCGGCAGCAGATTCTGTTGAGTCAGCTGG |
| | | 5′ CTCAACAGAATCTGCTGCCGCTGCTTTTTCAAGATAAACATCAAAATGGC |
| | SEQ_0933$^{GPGP}$ | 5′ GATGTTTATCTTGAAAAAGGTCCTGGTCCTGATTCTGTTGAGTCAGCTGG |
| | | 5′ CTCAACAGAATCAGGACCAGGACCTTTTTCAAGATAAACATCAAAATGGC |
| | SEQ_0933 cloning primer | 5′ GTCGTCGAATTCTGTTGATGGCAATTTGTTGGTG |
| **SEQ_2190** | SEQ_2190 | 5′ GTCGTCGGATCCTTGATATGAAAAGCGATGCCAAAAAGG |
| | SEQ_2190$^{ala}$ | *5′* GATGTTTATCTTGAAAAAGCAGCGGCAGCAGATATGAAAAGCGATGCCAAAAAGG |
| | | 5′ GCTTTTCATATCTGCTGCCGCTGCTTTTTCAAGATAAACATCAAAATGGC |

| | | |
|---|---|---|
| | SEQ_2190$^{GPGP}$ | 5′ GATGTTTATCTTGAAAAAGGTCCTGGTCCTGATATGAAAAGCGATGCCAAAAAGG |
| | | 5′ GCTTTTCATATCAGGACCAGGACCTTTTTCAAGATAAACATCAAAATGGC |
| | SEQ_2190 cloning primer | 5′ GTCGCTGAATTCAGTTGCTGGTAATTGTTTTTTAGG |
| **SEM** | SeM | 5′ GTCGTCGGATCCTTAACTCTGAGGTTAGTCGTACGGGATGCTAAGGTAGCAGAGCTTG |
| | SeM$^{ala}$ | 5′ GATGTTTATCTTGAAAAAGCAGCGGCAGCAAACTCTGAGGTTAGTCGTACGG |
| | | 5′ AACCTCAGAGTTTGCTGCCGCTGCTTTTTCAAGATAAACATCAAAATGGC |
| | SeM$^{GPGP}$ | 5′ GATGTTTATCTTGAAAAAGGTCCTGGTCCTAACTCTGAGGTTAGTCGTACGG |
| | | 5′ AACCTCAGAGTTAGGACCAGGACCTTTTTCAAGATAAACATCAAAATGGC |
| | SeM cloning primer | 5′ GACGACGAATTCAGATGGCAATTGACCTGCTTTAGC |
| **SEQ_0232** | SEQ_0232 | 5′ GTCGTCGGATCCTTTTGAAAGCAGAAACAACTCAATTG |
| | SEQ_0232$^{ala}$ | 5′ GATGTTTATCTTGAAAAAGCAGCGGCAGCATTGAAAGCAGAAACAACTCAATTG |
| | | 5′ GTTTCTGCTTTCAATGCTGCCGCTGCTTTTTCAAGATAAACATCAAAATGGC |
| | SEQ_0232$^{GPGP}$ | 5′ GATGTTTATCTTGAAAAAGGTCCTGGTCCTTTGAAAGCAGAAACAACTCAATTG |
| | | 5′ GTTTCTGCTTTCAAAGGACCAGGACCTTTTTCAAGATAAACATCAAAATGGC |
| | SEQ_0232 cloning primer | 5′ GTCGCTGAATTCTGATGGTAGCTGGCCTGCTG |

[1] BamHI (GGATCC) and EcoRI (GAATTC) restriction sites are underlined in the sequences.[2] Sequences of the peptide linkers are shown in red (tetra alanine) and blue (glycine-proline-glycine-proline). * SDM = Site directed mutagenesis.

International Journal of *Molecular Sciences*

**Table S2.** RT-qPCR reaction primers.

| | Primers Sequence |
|---|---|
| ACTB | 5′ CCAGCACGATGAAGATCAAG<br>5′ GTGGACAATGAGGCCAGAAT |
| GAPDH | 5′ CAGAACATCATCCCTGCTTC<br>5′ ATGCCTGCTTCACCACCTTC |
| IFNγ | 5′ CTACCTATTACTGCCAGGCCG<br>5′ TCCAGGAAAAGAGGCCCAC |
| TNFα | 5′ AAAGGACATCATGAGCACTGAAAG<br>5′ GGGCCCCCTGCCTTCT |
| il-10 | 5′ GCCTTGTCGGAGATGATCCA<br>5′ TTTTCCCCCAGGGAGTTCAC |
| il-6 | 5′ TGCTGGCTAAGCTGCATTCA<br>5′ GGAAATCCTCAAGGCTTCGAA |

```
SpeS    MKRLTFILIIL---MVMSTTNLNAVNSESHKDISTIKSKLLYAYTITPYDYKNCDVIFIT  57
        MK++  I I+    +++ +T    + S+S KDIS +KS LLYAYTITPYDYK+C V F T
SpeC    MKKINIIKIVFTITVILISTISPIIKSDSKKDISNVKSDLLYAYTITPYDYKDCRVNFST  60
                                                 YAY

                                                           PS00277
SpeS    THTLNIDTQKYKGQCYYINSEVDSEAAKKFKPGDKVDVFGLFYVLNSHTGEYIYGGITPS  117
        THTLNIDTQKY+G+ YYI+SE+  EA++KFK  D VDVFGLFY+LNSHTGEYIYGGITP+
SpeC    THTLNIDTQKYRGKDYYISSEMSYEASQKFKRDDHVDVFGLFYILNSHTGEYIYGGITPA  120
                                                           YGG*Txx
                                                            (LIV)
                                         PS00278
SpeS    QKNKVSKKLLGTLFVSGEPQKSLNNEITLEKDLITIQEFDFKIRNYLMKKYNLYSTTSPY  177
        Q NKV+ KLLG LF+SGE Q++LNN+I LEKD++T QE DFKIR YLM  Y +Y  TSPY
SpeC    QNNKVNHKLLGNLFISGESQQNLNNKIILEKDIVTFQEIDFKIRKYLMDNYKIYDATSPY  180
        xxN                           Kxx*xxxx*DxxxRxxLxxxxx*Y
                                      (LIVF)(LIVF)      (LIV)

SpeS    KGGRIEIGMKDGKHEKIDLFFFPNKGTREDIFKKYKDNKTLDMKLFSHFDVYLEK  232
          GRIEIG KDGKHE+IDLF  PN+GTR DIF KYKDN+ ++MK FSHFD+YLEK
SpeC    VSGRIEIGTKDGKHEQIDLFDSPNEGTRSDIFAKYKDNRIINMKNFSHFDIYLEK  235
                                                 HFD
```

**Figure S1.** Alignment of the predicted amino acid sequence of SpeS and SpeC. The conserved T cell receptor ß-binding motif YAY of SpeC [19,20], the consensus sequence Y-G-G-(LIV)-T-$x_4$-N and K-$x_2$-(LIVF)-$x_4$-(LIVF)-D-$x_3$-R-$x_2$-L-$x_5$-(LIV)-Y of the superantigen Prosite domains PS00277 and PS00278, respectively, and the zinc binding domain, HxD, are highlighted. Consensus matching residues are indicated in red. * represents one of the 3-4 possible amino acids indicated in brackets on the line directly below.

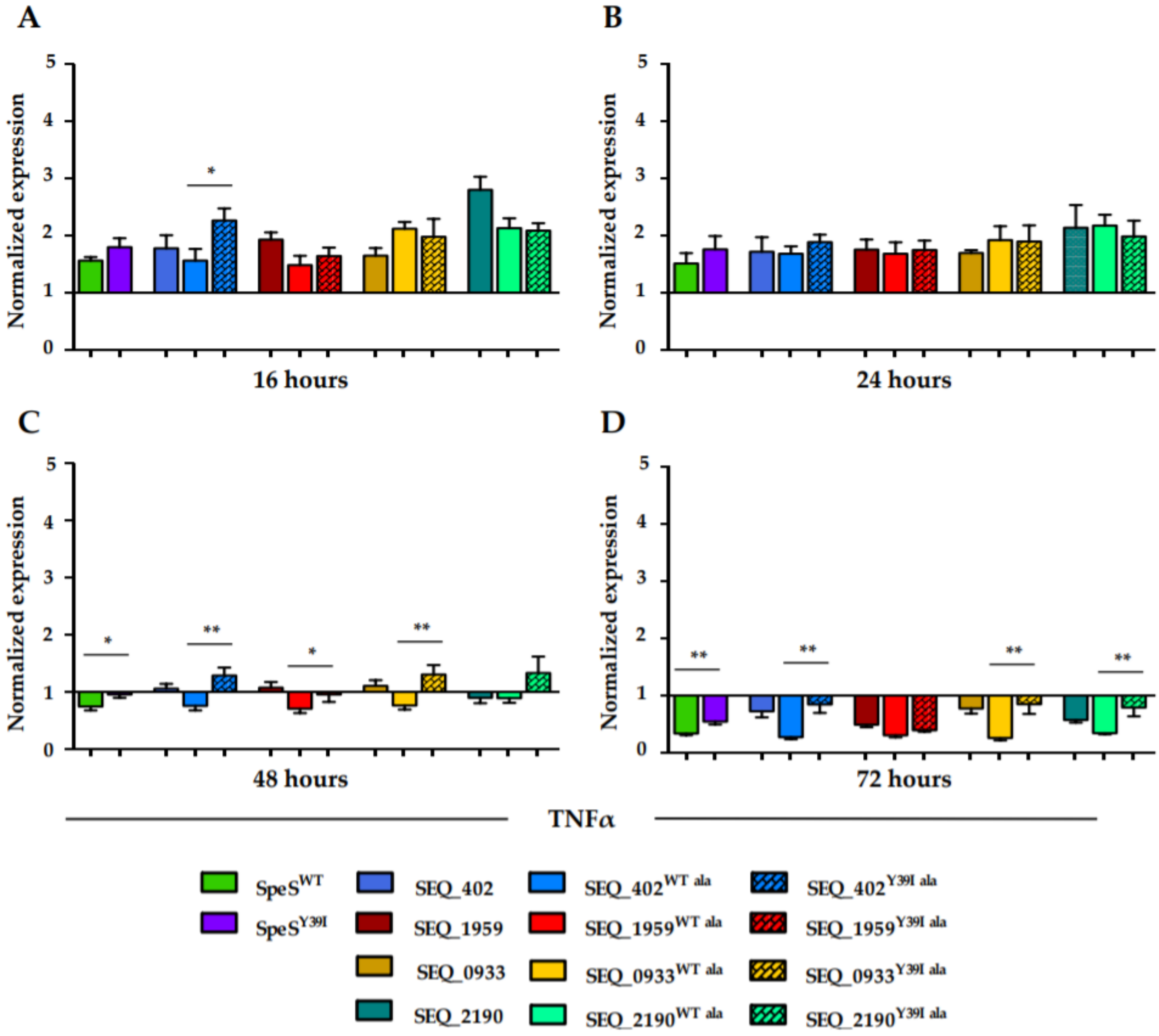


**Figure S2.** Expression of TNF*α* by qPCR. A total of 4 × $10^6$ equine PBMCs were stimulated in duplicate with 1 µg/ml of PBS (control), $SpeS^{WT}$, $SpeS^{Y39I}$ or the fusion proteins for (**A**) 16 h, (**B**) 24 h, (**C**) 48 h and (**D**) 72 h. Samples were normalized to the reference genes ß-actin and GAPDH and differences between the experimental samples and controls calculated. Data is from 3 independent experiments using PBMCs from 2 different donors. Error bars represent SEM. * $p \leq 0.05$, ** $p \leq 0.01$.

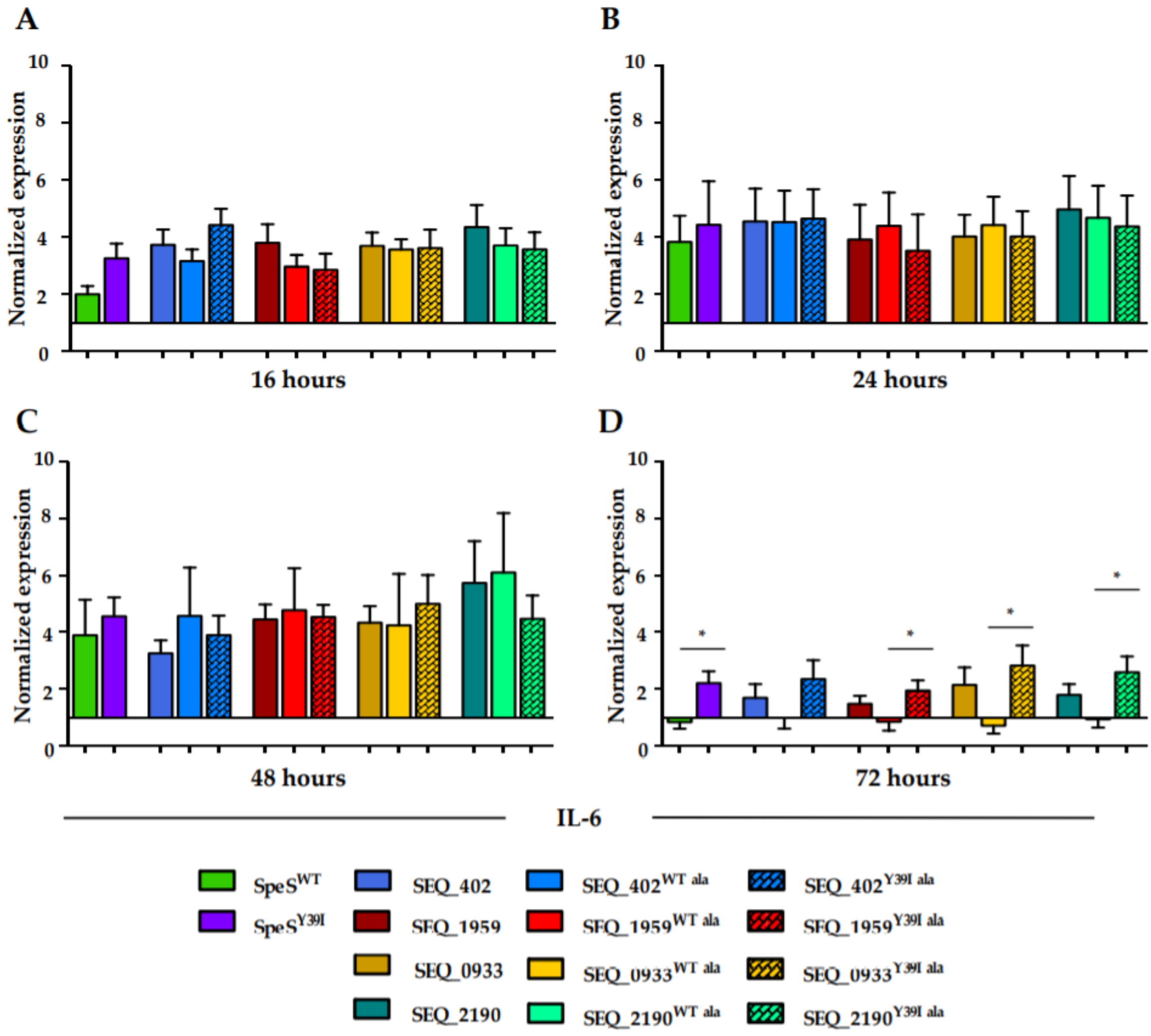


**Figure S3.** Expression of IL-6 by qPCR. Equine PBMCs (4 × $10^6$ cells/ml) were incubated in duplicate for (**A**) 16 h, (**B**) 24 h, (**C**) 48 h and (**D**) 72 h with 1 µg/mL of PBS (control), SpeS$^{WT}$, SpeS$^{Y39I}$ or the fusion proteins. The housekeeping genes, ß-actin and GAPDH, were used to normalize samples and differences between the experimental samples and the control samples were adjusted. Results are from 3 different experiments where PBMCs from 2 different donors were used. Error bars correspond to SEM. * $p \leq 0.05$.

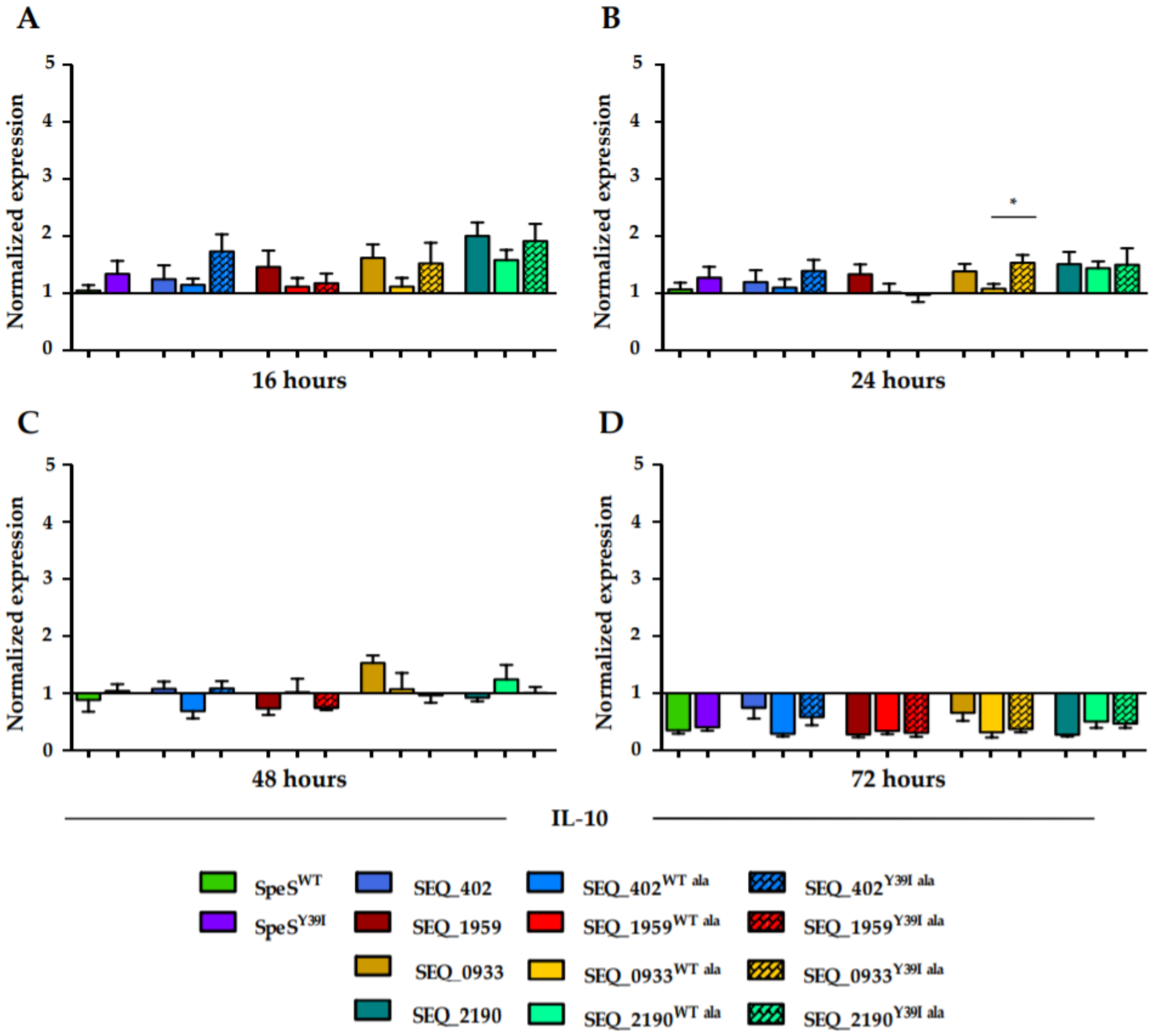


**Figure S4.** Expression of IL-10 by qPCR. A concentration of 4 × $10^6$ equine PBMCs were cultured in duplicate with 1 µg/mL of PBS (control), $SpeS^{WT}$, $SpeS^{Y39I}$ or the fusion proteins for (**A**) 16 h, (**B**) 24 hours, (**C**) 48 h and (**D**) 72 h. Samples were normalized against the reference genes β-actin and GAPDH and differences between the experimental samples and controls calculated. Data is from 3 independent experiments using PBMCs from 2 different donors. Error bars represent SEM. * $p \leq 0.05$.
